# Individual circulating microRNAs and continuous cognitive performance in a mixed inpatient-outpatient geriatric cohort: A cross-sectional pilot study

**DOI:** 10.1371/journal.pone.0357295

**Published:** 2026-09-11

**Authors:** Krzysztof Wilczyński, Wojciech Garczorz, Agnieszka Kosowska, Tomasz Francuz, Katarzyna Antoniak-Sobczak, Joanna Dudzińska-Griszek, Agnieszka Batko-Szwaczka, Jan Szewieczek

**Affiliations:** 1 Department of Geriatrics, School of Health Sciences in Katowice, Medical University of Silesia, Katowice, Poland; 2 Department of Biochemistry, School of Medicine in Katowice, Medical University of Silesia, Katowice, Poland; Nathan S Kline Institute, UNITED STATES OF AMERICA

## Abstract

**Background:**

Circulating microRNAs have been proposed as minimally invasive markers of cognitive impairment and neurodegenerative disease, but evidence for individual miRNAs in heterogeneous older populations remains limited.

**Objective:**

We examined cross-sectional associations between eight circulating serum miRNAs and continuous cognitive performance in a mixed inpatient-outpatient geriatric cohort.

**Methods:**

This cross-sectional analysis included 65 enrolled participants from a single-center, non-consecutive, mixed inpatient-outpatient geriatric cohort; 63 formed the analytic cohort (32 inpatients, 31 outpatients). Eligibility required written informed consent, availability of a routine-care Mini-Mental State Examination (MMSE) score, administration of Addenbrooke’s Cognitive Examination III (ACE-III), fasting serum sampling, and structural brain MRI during the same visit or within 48 hours. Eight predefined serum miRNAs were quantified by RT-qPCR with exogenous spike-in controls, with cel-miR-39-3p used as the normalization reference. The primary outcome was continuous MMSE score. Secondary outcomes were ACE-III total score, the ACE-III attention/orientation subscale, and threshold-defined MMSE and ACE-III impairment categories. Multivariable linear or logistic regression models were adjusted for age, sex, years of education, and recruitment setting. The main analysis used adjusted linear regression for continuous MMSE with Benjamini-Hochberg false discovery rate correction across the eight individual-miRNA MMSE models. Bootstrap was used to assess the stability of the MMSE estimates. Sensitivity analyses for the three strongest MMSE associations included Huber robust regression and miRNA by setting interaction testing.

**Results:**

In multivariable models adjusted for age, sex, years of education, and recruitment setting, higher levels of miR-132, miR-545, and miR-34a were linked to lower MMSE scores. A 1-SD increase in each marker corresponded to an MMSE decrease of about 1.1–1.3 points. These were the strongest single-miRNA findings in the dataset and met the prespecified FDR threshold in the primary models, although bootstrap analyses showed limited precision. Robust regression gave the same negative pattern for all three markers, suggesting that the results were not driven by a small number of extreme observations. Evidence of between-setting differences was seen only for miR-132 and only at the nominal level. No individual miRNA showed a clear nominal association with ACE-III total, and the ACE-III attention/orientation and MRI-adjusted analyses were exploratory. In secondary threshold-based models, no single miRNA remained significant after FDR correction.

**Conclusions:**

In this cross-sectional pilot cohort, higher miR-132, miR-545, and miR-34a were associated with lower MMSE scores in adjusted analyses. However, effect sizes were small, bootstrap analysis showed considerable uncertainty, and the findings are exploratory and hypothesis-generating. These results require evaluation in larger consecutive cohorts and do not establish diagnostic, prognostic, or standalone clinical biomarker utility.

## Introduction

Cognitive impairment is common in older adults seen in both geriatric wards and outpatient clinics, and timely recognition is important for decision-making capacity, medication safety, discharge planning, rehabilitation, and referral for more specific etiologic work-up [1–3]. Contemporary diagnostic pathways integrate clinical assessment, cognitive testing, structural imaging, and, when available, disease-anchored biomarkers, but these pathways can be difficult to deliver consistently in routine geriatric practice, particularly in multimorbid inpatients and resource-constrained outpatient settings [1–3]. Interest in minimally invasive blood-based biomarkers has therefore intensified.

Circulating microRNAs are attractive candidates because they are stable in blood matrices, measurable by targeted RT-qPCR workflows, and linked to neuronal, vascular, inflammatory, and systemic aging processes [4–8]. At the same time, translation has been slowed by substantial heterogeneity in cohort selection, reference standards, pre-analytical handling, normalization, and analytical pipelines [8–12]. Reviews and meta-analyses repeatedly conclude that the literature contains promising signals but limited reproducibility, especially when small discovery studies are over-interpreted or when marker sets are not externally validated [9–12].

Several of the miRNAs examined in the present cohort have plausible links to neurodegeneration or cognitive decline. MiR-132 is closely connected to synaptic plasticity and tau-related biology and has repeatedly emerged in Alzheimer-related literature [10,12,13]. MiR-34a is implicated in stress signaling and apoptosis and has also been reported among circulating biomarker candidates in Alzheimer’s disease [12,14–16]. MiR-545 has appeared in prior plasma biomarker work, but published findings also highlight the problem of between-cohort variability [15]. Whether such individual signals remain detectable in a clinically heterogeneous geriatric cohort spanning inpatient and outpatient settings is uncertain.

Beyond these better-studied candidates, the remaining study miRNAs also map onto disease-relevant biology, although human dementia literature is much less even. MiR-125b has been linked to tau phosphorylation and cognitive deficits in experimental models, miR-146a and miR-181a to inflammatory and progression-related mechanisms in prodromal Alzheimer’s disease, and miR-181c to plasma signatures related to synaptic dysfunction or microglial amyloid handling. By contrast, miR-532-5p and miR-545-3p remain comparatively underexplored in dementia cohorts, even though both have appeared in blood-based or mechanistic studies relevant to vascular or blood-brain barrier biology. This mix of biologically mature and relatively sparse candidates is useful for interpretation: concordance with prior work is more expected for miR-132 and miR-34a, whereas signals involving miR-532-5p or miR-545-3p are potentially more novel but also require greater caution [16–27].

We focused this study on continuous cognition in a geriatric biomarker cohort. Rather than examining multi-marker threshold classification, we evaluated whether predefined serum miRNAs were associated with MMSE as the primary outcome, with ACE-III total, ACE-III attention/orientation, and threshold-defined impairment outcomes analyzed secondarily. We expected any single-marker association to be modest and more detectable for continuous cognition than for threshold-defined states.

## Methods

### Study design and setting

This was a single-center, cross-sectional pilot geriatric biomarker study performed at a tertiary-care academic medical hospital between the 7th of July 2021 and the 28th of August 2023. Participants were recruited from two clinical settings: an academic geriatric inpatient ward and an affiliated geriatric outpatient clinic. Recruitment was non-consecutive and conducted during prespecified on-site study coverage periods when a trained investigator was available. During these periods, eligible elective inpatients and clinic attendees were screened from daily admission or appointment lists and approached in person. Recruitment was intermittently paused because of COVID-19 restrictions and investigator unavailability. Outside coverage periods, potentially eligible patients were not systematically screened or approached. Some eligible patients declined participation and no incentives were provided; therefore, the sample represents a convenience sample rather than a consecutive series. A screening log was not maintained, so the numbers screened, eligible, approached, and declining participation were not prospectively captured; participant flow is therefore reported from enrolled cases forward. The study was observational and non-interventional, and all procedures were completed during a single study visit or within 48 hours. The study protocol was reviewed and approved by the Bioethics Committee of the Medical University of Silesia in Katowice, Poland (original approval no. KNW/0022/KB1/43/18, 15 May 2018). Subsequent approvals/amendments were issued on 1 October 2019 (PCN/0022/KB1/43/I/18/19), 21 February 2020 (PCN/0022/KB1/43/II/18/19/20), and 20 September 2022 (PCN/CBN/0052/KB1/43/VI/18/19/20/21/22). The 20 September 2022 approval extended the approved study period to 30 September 2027 and approved an increase in planned enrolment to 65 participants. The recruitment period for the present analysis (7 July 2021 to 28 August 2023) was covered by these approvals. Written informed consent was obtained from all participants before any study-specific procedures. The study was conducted in accordance with the Declaration of Helsinki. This design and the requirement for written consent, an available routine-care MMSE score, ACE-III assessment, blood sampling, and MRI within 48 hours improved procedural consistency, but also limited representativeness of frailer older adults and those with more advanced dementia.

No formal a priori power calculation was used to define a definitive hypothesis-testing sample size. This was a budget-constrained pilot biomarker study supported by institutional grant funding, with recruitment and laboratory processing planned for approximately 60 participants. The final analytic sample of 63 participants reflected available grant resources, laboratory capacity, availability of a routine-care MMSE score, administration of ACE-III, fasting blood sampling, and MRI within the study window. Consequently, the study was powered only to detect relatively large single-marker associations and was underpowered for sparse binary outcomes, particularly dementia-range MMSE impairment. Negative findings, particularly in threshold-defined models with sparse events, should therefore be interpreted as inconclusive rather than as evidence of no association.

### Participants

A total of 65 participants consented/enrolled; 63 had complete core data for the analytic cohort, including 32 inpatients and 31 outpatients. Complete-case models were fitted separately for each individual miRNA and outcome combination; therefore, the effective sample size varied slightly across markers because marker-specific assay availability differed in the analysis dataset, and the final modeled n for each miRNA reflected complete-case availability for that miRNA-outcome-covariate set.

Eligible participants were elective geriatric inpatients or outpatients aged 60 years or older who were able to provide written informed consent, had an available routine-care MMSE score, and completed ACE-III assessment, venous blood sampling, and structural brain MRI within 48 hours, typically on the same day. Exclusion criteria in the study protocol included severe systemic or acute illness precluding assessment, major depressive episode, Parkinson’s disease or other neurodegenerative movement disorders, known advanced dementia precluding informed consent or completion of study procedures, territorial stroke or intracranial mass lesion on MRI, MRI contraindications, acute admission via the emergency department, and inability to perform frailty testing safely. At enrolment, a structured medical history, physical examination and comprehensive geriatric assessment were performed as part of standard admission protocols.

### Clinical and cognitive assessment

The study used structured clinical data, the routine-care MMSE score, ACE-III cognitive assessment, functional assessment, fasting venous blood sampling, and structural brain MRI obtained within 48 hours of one another. Functional assessment included the Barthel Index and Lawton Instrumental Activities of Daily Living. ACE-III examiners were blinded to laboratory data. Laboratory personnel involved in RNA extraction and RT-qPCR, and analysts generating expression variables from crossing-point (Cp) data, were blinded to the routine-care MMSE score, ACE-III results, and clinical classification.

Cognitive assessment included use of the Mini-Mental State Examination (MMSE; range 0–30) score recorded after routine clinical administration as part of standard geriatric care, and administration of the Addenbrooke’s Cognitive Examination III (ACE-III; range 0–100) as part of the study assessment. Thus, the present analysis used the available routine-care MMSE score, whereas ACE-III was administered as part of the study assessment. Threshold-defined impairment outcomes were defined as ACE-III ≤ 88 for any impairment (CI-ACE), ACE-III ≤ 82 for dementia-range impairment (Dementia-ACE), MMSE ≤ 26 for any impairment (CI-MMSE), and MMSE ≤ 23 for dementia-range impairment (Dementia-MMSE). In the current analysis, the primary outcome was continuous MMSE score. MMSE was selected as the main continuous endpoint because it was available across the full cohort, is widely used in routine geriatric care, and allowed a clearly defined primary testing family; ACE-III total was retained as secondary contextual analysis. Secondary outcomes were continuous ACE-III total score, the ACE-III attention/orientation subscale, and the threshold-defined MMSE and ACE-III outcomes used elsewhere in the manuscript. These threshold-defined outcomes were based on screening-test cut-offs and should not be interpreted as adjudicated clinical diagnoses, etiologic dementia classifications, or biomarker-confirmed disease states.

### Blood sampling, RNA extraction, and RT-qPCR

After an overnight fast, venous blood was collected between 07:00 and 10:00, processed to serum, centrifuged within 60 minutes at 2,000 g for 10 minutes at 4 °C, aliquoted, and stored at −80 °C. Samples with visible hemolysis were redrawn when feasible; if redraw was not possible, the specimen was discarded and excluded from analysis. Total RNA, including small RNAs, was extracted from 200 µL serum using the miRNeasy Serum/Plasma Advanced Kit (Qiagen) according to the manufacturer’s instructions. After addition of RPL buffer, external spike-in controls were added to each sample (0.002 fmol cel-miR-39-3p and 0.15 fmol UniSp6) to monitor extraction and RT-qPCR performance. Cel-miR-39-3p was selected as the normalization reference because it was added exogenously to all samples and allowed monitoring of extraction and RT-qPCR performance. Endogenous reference miRNAs or global mean normalization were not used because the study quantified a limited predefined eight-miRNA panel and no stable endogenous serum reference miRNA had been prespecified for this cohort.

Reverse transcription was performed using the miRCURY RT Kit (Qiagen), and RT-qPCR was performed using the miRCURY LNA SYBR Green PCR Kit (Qiagen) on a LightCycler 480 platform (Roche) with 45 amplification cycles. No-template controls were processed in parallel. Melting-curve analysis was used to confirm a single specific product. All reactions were run in duplicate. Duplicate pairs with poor amplification quality or non-specific melt curves were excluded, and runs with duplicate coefficient of variation greater than 5% were repeated where possible. The RT-qPCR workflow was aligned with MIQE principles.

### miRNA definition and preprocessing

The study examined eight predefined individual miRNAs: miR-146a-5p, miR-181a-5p, miR-34a-5p, miR-545-3p, miR-125b-5p, miR-532-5p, miR-132-3p, and miR-181c-5p. The eight miRNAs were selected a priori based on prior Alzheimer’s disease/cognitive impairment literature, biological plausibility, and availability within the laboratory panel. MiR-132 and miR-34a were considered better-supported candidates because of links to synaptic/tau biology and stress/apoptotic signaling. MiR-545 was included because of prior plasma biomarker reports but was regarded as less consistently validated. MiR-125b, miR-146a, miR-181a, and miR-181c were selected because of reported links to tau biology, neuroinflammation, disease progression, synaptic dysfunction, or microglial/mitochondrial pathways. MiR-532 was considered exploratory because dementia-specific human evidence remains limited.

The de-identified participant-level dataset and accompanying variable dictionary are provided as S1 Data and S2 Data Dictionary.

Primary exposure variables were the cel-miR-39-normalized expression measures. Normalization was computed as ΔCp = Cp(target) – Cp(cel-miR-39-3p). Relative expression was derived using the 2^-ΔCp approach. Reactions with Cp values  ≥ 39 or no amplification were classified as non-detects. Cp  ≥ 39 was used as the non-detect threshold because amplification at or beyond this late-cycle threshold was not considered reliably quantifiable. Participant-level expression values were calculated from available valid target/cel-miR-39-normalized duplicate ratios; if only one valid duplicate ratio was available, that value was retained, and participant-level miRNA values were set missing only when no valid normalized duplicate ratio was available after quality control. Participant-level cel-miR-39-normalized expression values were then winsorized at the pre-winsorization mean ±3 sample standard deviations for each miRNA. Winsorization at ±3 sample SD was used to limit the leverage of extreme participant-level expression values while retaining observations in this small pilot dataset. Values above the upper threshold were set to the upper threshold; no values fell below the lower threshold. The winsorized expression variables were then z-standardized before modelling. Final analytic participant duplicate-reaction non-detect counts (126 duplicate reactions per miRNA) and participant-level winsorization counts are reported in S1 Table.

### MRI review

All participants underwent structural brain MRI during the study visit. MRI review was performed blinded to the routine-care MMSE score, ACE-III results, and miRNA measurements. Radiology reports were reviewed by a study physician to confirm absence of major alternative intracranial pathology that could independently explain cognitive impairment, including territorial infarction, intracranial tumor, or normal-pressure hydrocephalus. White-matter hyperintensities were rated using Fazekas scores for descriptive characterization. Gray-matter, brain, and CSF fractions derived from volumetric measurements were available for sensitivity analyses but were not used in the primary models.

### Statistical analysis

Analyses were performed in Python 3.11 using statsmodels and scikit-learn. Age and years of education were treated as continuous covariates and z-standardized before modelling. Years of education were derived from reported educational attainment using the conversion: primary=7 years, vocational=9 years, secondary=12 years, and higher=16 years. Sex was coded as 0 for female and 1 for male. Recruitment setting was coded as inpatient versus outpatient and retained in the adjusted models because the two source populations differed clinically and cognitively at baseline.

The analytic cohort comprised 63 participants with complete core data. Complete-case modelling was performed separately for each miRNA-outcome combination. Thus, the reported sample size for each model corresponds to the number of participants with non-missing data for the specific miRNA, outcome, and covariates included in that model. No imputation of missing participant-level miRNA variables or cognitive variables was performed. The main analysis consisted of adjusted linear regression of continuous MMSE on each individual cel-miR-39-normalized miRNA, with age, sex, years of education, and recruitment setting included as covariates. Regression coefficients are reported per 1-standard-deviation higher miRNA level. Panel-based analyses of threshold-defined impairment from this cohort are reported separately in a submitted manuscript.

For the primary endpoint, p-values from the eight individual-miRNA MMSE models were adjusted using the Benjamini-Hochberg false discovery rate procedure. ACE-III total was reported with nominal p values only, whereas false discovery rate adjustment was retained for secondary eight-test families, including ACE-III attention/orientation and the threshold-defined MMSE and ACE-III outcomes. For the MMSE models, 2,000 case-resampling bootstrap samples with replacement were drawn and the full adjusted model was refit in each resample to generate percentile 95% confidence intervals. The three strongest MMSE associations were also refit using Huber robust regression as a sensitivity analysis. To assess possible case-mix effects, miRNA by setting interaction testing was performed for the same three markers. Reporting was informed by the STROBE guideline for cross-sectional studies.

## Results

### Study population

**Fig 1:**
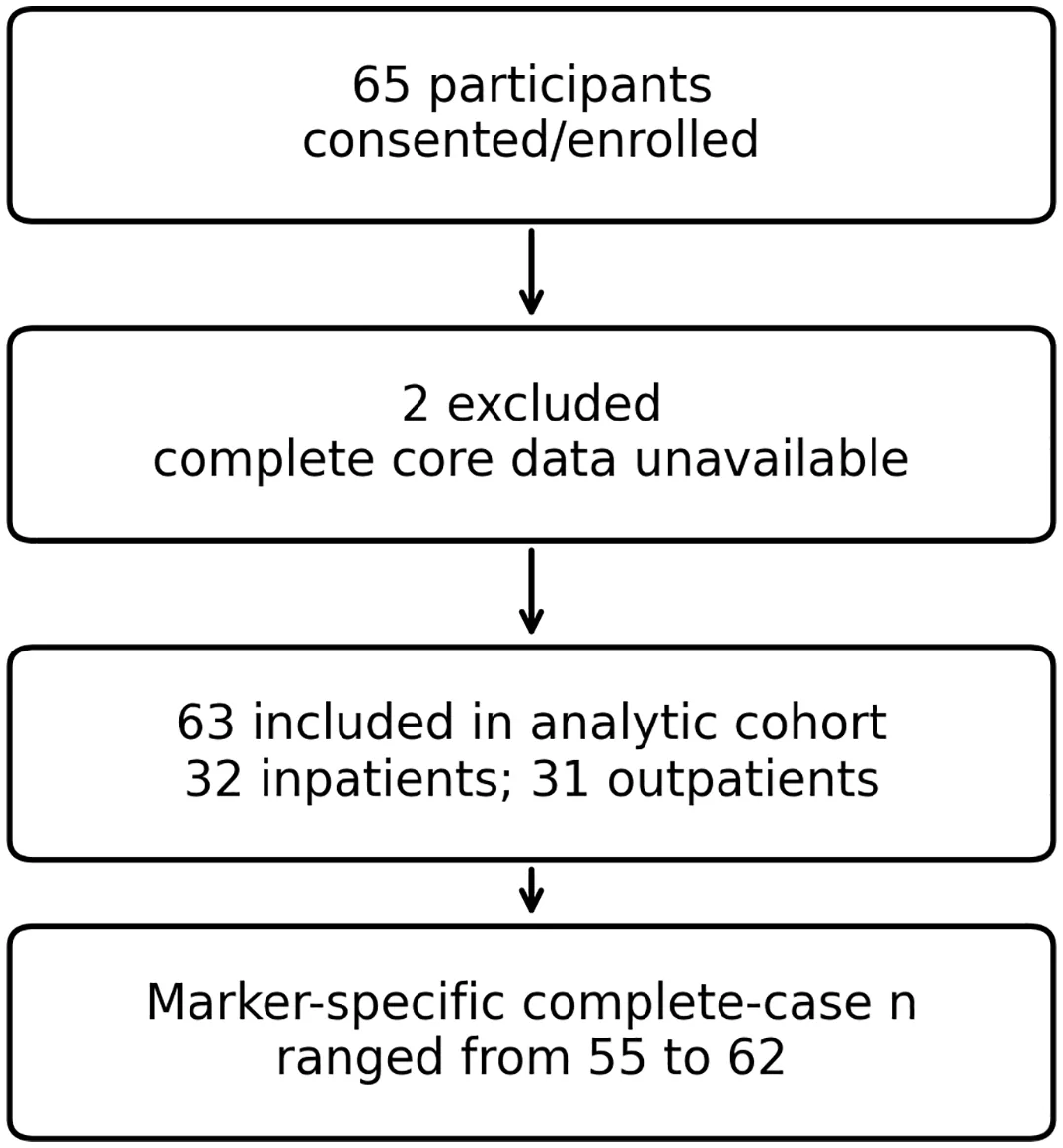
Participant flow.

A total of 65 participants were enrolled; two consented participants were excluded because complete core data for the present analyses were unavailable. Participant flow is summarized in Fig 1. The final cohort comprised 63 participants (46 women, 17 men), including 32 inpatients and 31 outpatients. Because a formal screening log was not maintained, denominator data for screening and eligibility were unavailable; participant flow is therefore reported from the enrolled cohort onward. Individual-miRNA assay availability ranged from 55 participants for miR-545–62 for miR-181c, and complete-case model-specific n varied accordingly across miRNA-outcome-covariate sets. Mean age was 69.1 ± 3.9 years, mean education was 12.2 ± 2.3 years, mean MMSE was 27.2 ± 3.7, and mean ACE-III total score was 87.6 ± 12.7. Screening-threshold-defined impairment frequencies were 24/63 (38.1%) for CI-ACE, 15/63 (23.8%) for Dementia-ACE, 16/63 (25.4%) for CI-MMSE, and 7/63 (11.1%) for Dementia-MMSE. Inpatients were older than outpatients, had fewer years of education, and had lower MMSE and ACE-III scores (Table 1). The Charlson Comorbidity Index was also modestly higher in inpatients, whereas sex distribution did not differ materially.

**Table 1:** Baseline characteristics, education level distribution, and miRNA assay availability of the analytic cohort by recruitment setting.

Characteristic	Overall (n = 63)	Inpatients (n = 32)	Outpatients (n = 31)	p
**Age, years**	69.1 ± 3.9	70.7 ± 3.0	67.4 ± 4.0	<0.001
**Education, years**	12.2 ± 2.3	11.5 ± 2.5	12.9 ± 1.9	0.014
**Education level, n (%)**				0.019
**Primary**	1/63 (1.6%)	1/32 (3.1%)	0/31 (0.0%)	
**Vocational**	11/63 (17.5%)	10/32 (31.2%)	1/31 (3.2%)	
**Secondary**	38/63 (60.3%)	16/32 (50.0%)	22/31 (71.0%)	
**Higher**	13/63 (20.6%)	5/32 (15.6%)	8/31 (25.8%)	
**Male sex**	17/63 (27.0%)	8/32 (25.0%)	9/31 (29.0%)	0.782
**ACE-III total**	87.6 ± 12.7	82.6 ± 12.8	92.6 ± 10.7	0.001
**MMSE**	27.2 ± 3.7	26.1 ± 4.1	28.4 ± 2.9	0.012
**BMI, kg/m²**	26.6 ± 4.3	26.9 ± 4.4	26.2 ± 4.2	0.585
**CRP, mg/L**	5.0 [5.0-5.0]	5.0 [5.0-5.3]	5.0 [5.0-5.0]	0.001
**Creatinine, mg/dL**	0.9 ± 0.2	0.9 ± 0.2	0.8 ± 0.2	0.428
**eGFR, mL/min/1.73 m²**	76.1 ± 13.5	72.6 ± 14.9	80.0 ± 10.7	0.028
**Platelets, 10^3/µL**	248.9 ± 48.0	246.5 ± 44.4	251.4 ± 52.3	0.693
**Charlson Comorbidity Index**	2 [1 –2]	2 [2 –2]	2 [1 –2]	0.014
**ACE-III ≤ 88**	24/63 (38.1%)	20/32 (62.5%)	4/31 (12.9%)	<0.001
**ACE-III ≤ 82**	15/63 (23.8%)	14/32 (43.8%)	1/31 (3.2%)	<0.001
**MMSE ≤26**	16/63 (25.4%)	12/32 (37.5%)	4/31 (12.9%)	0.041
**MMSE ≤23**	7/63 (11.1%)	6/32 (18.8%)	1/31 (3.2%)	0.104
**miRNA assay availability, n (%)**				
**miR-125b**	61/63 (96.8%)	31/32 (96.9%)	30/31 (96.8%)	
**miR-132**	61/63 (96.8%)	30/32 (93.8%)	31/31 (100.0%)	
**miR-146a**	61/63 (96.8%)	31/32 (96.9%)	30/31 (96.8%)	
**miR-181a**	60/63 (95.2%)	30/32 (93.8%)	30/31 (96.8%)	
**miR-181c**	62/63 (98.4%)	31/32 (96.9%)	31/31 (100.0%)	
**miR-34a**	61/63 (96.8%)	31/32 (96.9%)	30/31 (96.8%)	
**miR-532**	61/63 (96.8%)	30/32 (93.8%)	31/31 (100.0%)	
**miR-545**	55/63 (87.3%)	27/32 (84.4%)	28/31 (90.3%)	

Continuous variables are reported as mean ± SD or median [IQR] as appropriate; categorical variables are n (%). For the education level rows, the p value corresponds to the overall distribution across categories by recruitment setting. BMI was available in 59 participants (30 inpatients, 29 outpatients). CRP was available in 62 participants (31 inpatients, 31 outpatients); creatinine, eGFR, and platelets were available in 62 participants (32 inpatients, 30 outpatients). Because individual-miRNA assay availability differed by marker, model-specific n for the adjusted regression analyses ranged from 55 to 62. For CRP, many values were recorded at the lower reporting limit of 5.0 mg/L; all nine observations above 5.0 mg/L occurred among inpatients, whereas no outpatient value exceeded 5.0 mg/L. Therefore, similar displayed medians and IQRs may coexist with a rank-based p value reflecting distributional differences outside the displayed quartiles.

### Individual miRNAs and continuous cognitive performance

**Fig 2:**
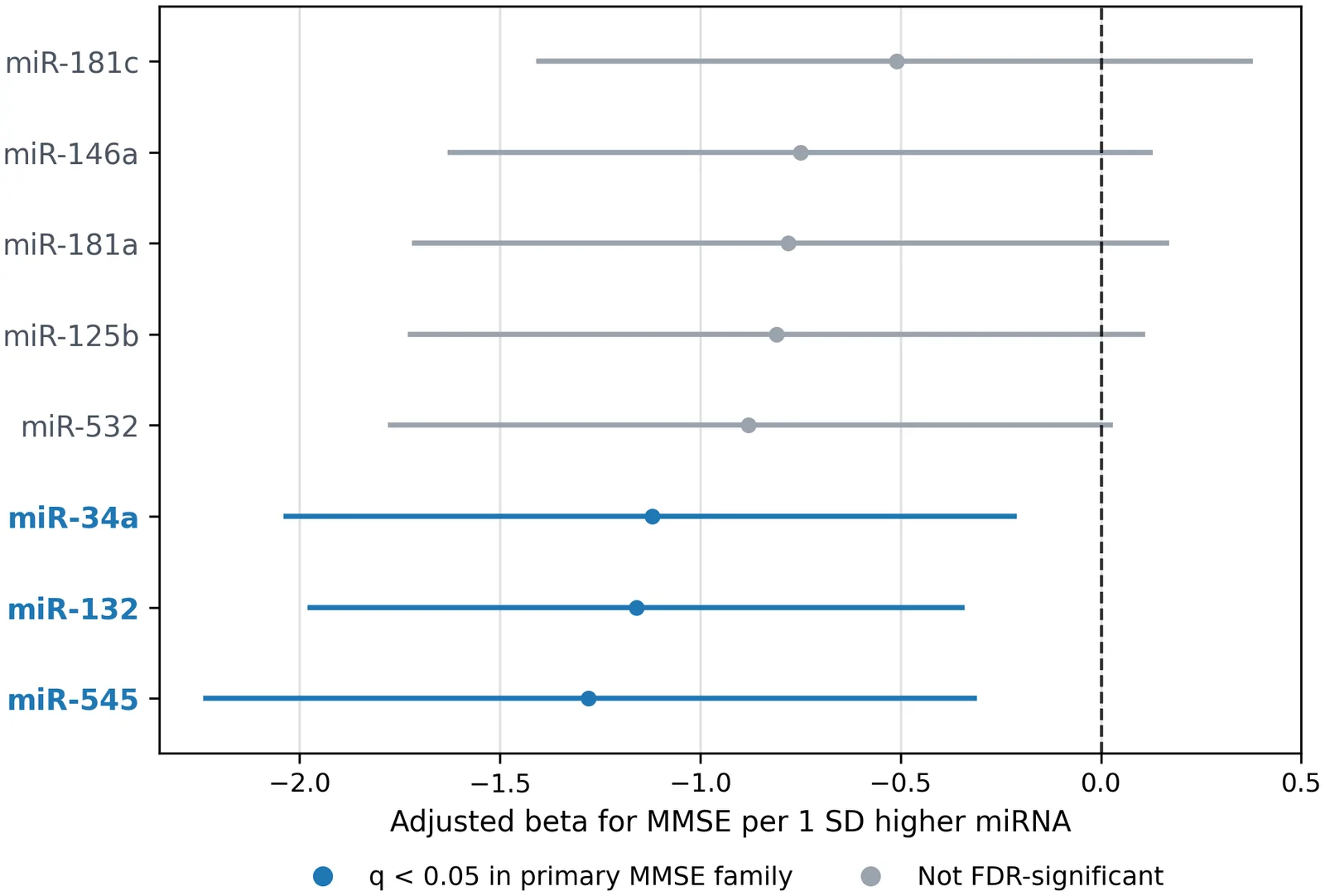
Forest plot of multivariable-adjusted associations between predefined serum miRNAs and MMSE.

In models adjusted for age, sex, years of education, and recruitment setting, higher levels of several individual cel-miR-39-normalized miRNAs were associated with lower MMSE scores (Table 2; Fig 2). Differences in analytic n across markers reflected marker-specific assay availability in the analysis dataset; final modeled n ranged from 55 for miR-545–62 for miR-181c. The strongest inverse associations were observed for miR-132 (beta −1.16 MMSE points per 1 SD, 95% CI −1.98 to −0.34; p = 0.006; q = 0.042; n = 61), miR-545 (beta −1.28, 95% CI −2.24 to −0.31; p = 0.010; q = 0.042; n = 55), and miR-34a (beta −1.12, 95% CI −2.04 to −0.21; p = 0.017; q = 0.045; n = 61). MiR-532 showed a similar inverse estimate that did not reach nominal significance (beta −0.88, p = 0.057) and did not survive FDR correction. The remaining miRNAs showed smaller and non-robust associations.

**Table 2:** Multivariable-adjusted associations of individual cel-miR-39-normalized miRNAs with continuous cognition.

miRNA	n	MMSE beta(95% CI)	p	q	Bootstrap 95% CI for MMSE	n	ACE-III total beta(95% CI)	p
**miR-132**	61	−1.16 (−1.98 to −0.34)	0.006	0.042	−2.21 to 0.16	61	−1.44 (−4.19 to 1.32)	0.301
**miR-545**	55	−1.28 (−2.24 to −0.31)	0.010	0.042	−2.17 to 0.13	55	−2.06 (−5.46 to 1.34)	0.228
**miR-34a**	61	−1.12 (−2.04 to −0.21)	0.017	0.045	−1.78 to 1.58	61	−1.35 (−4.50 to 1.80)	0.395
**miR-532**	61	−0.88 (−1.78 to 0.03)	0.057	0.114	−1.80 to 0.75	61	−0.83 (−3.90 to 2.23)	0.588
**miR-125b**	61	−0.81 (−1.73 to 0.11)	0.084	0.120	−1.94 to 0.88	61	−0.62 (−3.78 to 2.55)	0.698
**miR-146a**	61	−0.75 (−1.63 to 0.13)	0.094	0.120	−1.89 to 0.82	61	−0.41 (−3.43 to 2.61)	0.787
**miR-181a**	60	−0.78 (−1.72 to 0.17)	0.105	0.120	−1.78 to 1.01	60	−1.14 (−4.34 to 2.06)	0.478
**miR-181c**	62	−0.51 (−1.41 to 0.38)	0.256	0.256	−1.99 to 0.72	62	−1.39 (−4.35 to 1.57)	0.350

Abbreviations: ACE-III, Addenbrooke’s Cognitive Examination III; CI, confidence interval; FDR q, Benjamini-Hochberg false discovery rate adjusted p value; MMSE, Mini-Mental State Examination. In Table 2, q values apply to the primary MMSE family only; the ACE-III total block is shown with nominal p values for secondary context. Bootstrap intervals are presented as conservative stability checks and were not used as additional significance criteria.

Markers meeting q < 0.05 within the eight-test primary MMSE family are indicated separately from non-significant markers. Points indicate adjusted regression coefficients for continuous MMSE per 1-standard-deviation higher cel-miR-39-normalized miRNA level. Horizontal lines indicate model-based 95% confidence intervals from the adjusted regression models; bootstrap percentile intervals are reported in Table 2 and are not plotted. Models were adjusted for age, sex, years of education, and recruitment setting. Marker-specific modeled n values were: miR-181c, 62; miR-146a, 61; miR-181a, 60; miR-125b, 61; miR-532, 61; miR-34a, 61; miR-132, 61; and miR-545, 55. Negative coefficients indicate worse MMSE performance with higher circulating miRNA levels.

Bootstrap analyses for the MMSE models yielded similarly negative point estimates but wider percentile confidence intervals. For miR-132, the bootstrap 95% interval was −2.21 to 0.16; for miR-545, −2.17 to 0.13; and for miR-34a, −1.78 to 1.58.

In Huber robust regression, the MMSE associations remained negative for miR-132 (beta −1.58, 95% CI −2.11 to −1.04), miR-545 (beta −1.44, 95% CI −2.13 to −0.75), and miR-34a (beta −1.54, 95% CI −2.18 to −0.91). Formal miRNA by setting interaction testing for these three markers did not show a consistent interaction pattern: only miR-132 showed nominal evidence of a stronger inverse association in inpatients than outpatients (interaction beta −1.94, 95% CI −3.54 to −0.34; p = 0.018), whereas the corresponding interactions for miR-545 and miR-34a were not statistically significant.

No individual miRNA showed a clear nominal association with ACE-III total score. The largest effect estimate was again seen for miR-545 (beta −2.06 ACE-III points, 95% CI −5.46 to 1.34; nominal p = 0.228), followed by miR-132 (beta −1.44; nominal p = 0.301) and miR-181c (beta −1.39; nominal p = 0.350) (Table 2). Thus, the strongest individual-marker associations in this cohort were observed for continuous MMSE rather than for the global ACE-III total score.

### Exploratory ACE-III subdomain and MRI-context analyses

Exploratory ACE-III subdomain analyses suggested that any domain-level signal was concentrated in the attention/orientation section. In multivariable-adjusted models, higher miR-34a (beta −0.73, 95% CI −1.26 to −0.20; p = 0.008; q = 0.036), miR-132 (beta −0.65, 95% CI −1.14 to −0.15; p = 0.011; q = 0.036), miR-545 (beta −0.74, 95% CI −1.32 to −0.16; p = 0.013; q = 0.036), and miR-532 (beta −0.61, 95% CI −1.14 to −0.09; p = 0.024; q = 0.047) were each associated with lower attention/orientation scores.

Gray-matter fraction was added as an additional adjustment for the top four attention-associated miRNAs (Table 3). The attention/orientation associations remained negative after this adjustment, but these small-sample models were included only to show that the miRNA signal was not simply duplicating a structural atrophy measure.

**Table 3:** Attention/orientation and MRI-context models for selected MMSE-associated miRNAs.

miRNA	n	ACE-III attention/orientation beta (95% CI)	p	q	n with gray-matter fraction	miRNA beta after gray-matter adjustment (95% CI)	p
**miR-34a**	58	−0.73 (−1.26 to −0.20)	0.008	0.036	56	−0.66 (−1.29 to −0.04)	0.039
**miR-132**	58	−0.65 (−1.14 to −0.15)	0.011	0.036	56	−0.48 (−0.97 to 0.00)	0.051
**miR-545**	52	−0.74 (−1.32 to −0.16)	0.013	0.036	50	−0.59 (−1.18 to 0.00)	0.050
**miR-532**	58	−0.61 (−1.14 to −0.09)	0.024	0.047	56	−0.59 (−1.09 to −0.08)	0.024

Note: Gray-matter fraction models were adjusted for age, sex, years of education, recruitment setting, and gray-matter fraction. These small-sample models are provided as context and should not be interpreted as independent validation of the main MMSE findings.

### Secondary threshold-defined impairment outcomes

No individual miRNA remained significant after FDR correction for CI-ACE, ACE-III-defined dementia-range impairment, CI-MMSE, or MMSE-defined dementia-range impairment. Full adjusted threshold-model estimates for all eight markers across CI-MMSE, MMSE-defined dementia-range impairment, CI-ACE, and ACE-III-defined dementia-range impairment are provided in S2 Table. The strongest binary signals were seen for MMSE-defined dementia-range impairment, where miR-34a showed a nominal association (OR 3.78, 95% CI 1.03 to 13.93; p = 0.046; q = 0.101) while miR-545 showed a similar borderline estimate (OR 5.02, 95% CI 0.98 to 25.87; p = 0.054; q = 0.101), but these estimates were based on only seven MMSE-defined dementia-range events in the full cohort and were therefore considered unstable. Full panel-based analyses of threshold-defined cognitive impairment from this cohort are reported separately in a submitted manuscript; the present analysis includes threshold-defined outcomes only as secondary context for individual miRNAs. S2 Table is limited to individual-marker logistic-regression estimates and does not include composite-panel performance metrics.

## Discussion

In this single-center, non-consecutive, mixed inpatient-outpatient geriatric cohort, selected circulating miRNAs showed small inverse associations with continuous cognitive performance. After adjustment for age, sex, education, and recruitment setting, higher miR-132, miR-545, and miR-34a were associated with lower MMSE scores, whereas associations with ACE-III total were weaker and no individual miRNA remained FDR-significant in threshold-defined impairment models. The subdomain and MRI-adjusted analyses were exploratory and did not materially strengthen the main findings. This focus complements panel-based analyses of threshold-defined impairment from the same cohort reported separately. These findings remain exploratory and hypothesis-generating and do not establish diagnostic, prognostic, or standalone clinical biomarker utility.

Reviews and meta-analyses suggest that circulating miRNAs may carry useful information in the context of Alzheimer’s disease and cognitive impairment, but replication across cohorts remains difficult because of differences in case definition, disease stage, sample handling, normalization, and analytic strategy [9–12]. The pattern observed here is broadly consistent with prior blood-based reports for miR-132, miR-34a, and miR-545 [15,28]. Prior support is strongest for miR-132 and miR-34a [13,16–20,28]. MiR-132 has been linked to synaptic function, tau-related pathways, and neuroprotection [10,12,13], whereas miR-34a has been associated with cellular stress and p53-linked apoptotic signaling [14] and altered circulating levels in Alzheimer’s disease cohorts [15,16,29]. Evidence for miR-545 remains thinner and less consistent across centers [15]. Overall, the present results support biological plausibility, but not disease specificity or clinical use. Recent plasma-miRNA work across the Alzheimer’s disease continuum has also linked circulating miRNA profiles to central amyloid, tau, and neurodegeneration biomarkers; these associations support biological relevance but still require cautious clinical interpretation [30].

The remaining study miRNAs are best interpreted as biological context rather than as stand-alone correlates of cognitive scores in this dataset. Prior work has linked miR-125b, miR-146a, and members of the miR-181 family to tau biology, neuroinflammation, progression, or illness severity [21–25,27], while miR-532-5p has only limited dementia-related support at present [26]. These links support pathway-level relevance, but not necessarily usefulness as individual clinical markers. In a small, mixed inpatient-outpatient geriatric cohort, biologically plausible miRNAs may reflect broader disease-related processes, multimorbidity, or illness burden rather than showing a clear one-to-one relation with a cross-sectional cognitive score. The weak or non-robust single-marker associations observed here are therefore informative in their own right, because they show that mechanistic plausibility does not automatically translate into reproducible biomarker performance. These miRNAs may therefore be more useful as contextual or panel-level variables than as stand-alone correlates of cognition.

Continuous analyses provided more stable and interpretable estimates in this small cohort than threshold-defined models, in which sparse events limited precision and no individual miRNA survived FDR correction. Categorization may discard information and reduces power, which is especially problematic in a cohort of 63 participants when only a small number met MMSE-defined dementia-range thresholds. The continuous models retained graded variation across the cognitive spectrum, whereas logistic models were more sensitive to sparse events and produced imprecise odds ratios. For early translational work in small cohorts, continuous cognitive outcomes may therefore be more useful than screening cut-offs for ranking candidate markers.

ACE-III samples a broader range of domains and generally shows better discrimination than MMSE for milder cognitive impairment and dementia, including in Polish validation work [31,32]. In the present dataset, however, the clearest single-marker associations were observed for continuous MMSE. In a small heterogeneous cohort, this may reflect where modest effects were most detectable rather than any intrinsic advantage of MMSE. MMSE was the prespecified primary continuous endpoint and may have tracked the dominant gradient of global cognitive performance with less noise, whereas the broader ACE-III total score may have diluted weak associations. The exploratory attention/orientation and MRI-adjusted analyses were directionally consistent with the main findings, but because they were secondary analyses performed in the same cohort, they should not be interpreted as independent confirmation.

This study has several limitations. The sample size was modest because this was a budget-constrained pilot biomarker study planned around available grant resources and laboratory capacity rather than a definitive power calculation; therefore, negative findings, particularly in threshold-defined models with sparse events, should be interpreted as inconclusive rather than as evidence of no association. Recruitment was coverage-based rather than fully consecutive, no screening log was maintained, and no external validation cohort was available. The absence of an external validation cohort limits generalizability. Because participation required an available routine-care MMSE score, ACE-III assessment, blood sampling, and MRI within 48 hours, the analytic sample may under-represent frailer older adults and those with more advanced dementia. Marker-specific assay availability led to small differences in sample size across analyses. For individual miRNAs, participant-level values were missing when no valid normalized duplicate ratio was available; if non-detectability correlated with cognitive status, marker-specific complete-case analyses could be affected by missing-not-at-random bias. The cross-sectional design does not allow assessment of change over time or progression. Because inpatients differed from outpatients in age, education, comorbidity burden, and baseline cognition, residual case-mix effects may still have influenced pooled associations despite adjustment. We did not have a systematically coded reason for inpatient admission or outpatient referral available for analysis; therefore, residual confounding by clinical indication, acute/subacute geriatric syndromes, or reason for assessment cannot be excluded. Although pre-analytical handling and RT-qPCR quality control were standardized, circulating miRNA results remain sensitive to normalization strategy and related analytical choices. Reliance on an exogenous spike-in controls technical variation in extraction and amplification but does not fully account for differences in total circulating RNA content, hemolysis-related shifts, or biological variation in endogenous miRNA background. Therefore, the findings should be interpreted as cel-miR-39-normalized relative expression associations rather than absolute circulating miRNA abundance. Visual hemolysis assessment was performed, but dedicated spectrophotometric or hemolysis-marker adjustment was not available. Hemolysis is a recognized nuisance factor that can affect plasma miRNA levels and variance [33].

Strengths include near-contemporaneous phenotyping, a clinically relevant mixed geriatric population, blinded laboratory workflows and blinded ACE-III assessment, a MIQE-aware RT-qPCR pipeline, adjustment for recruitment setting, multiplicity control for the primary endpoint, and bootstrap-based sensitivity analysis.

## Conclusions

In this mixed inpatient-outpatient cross-sectional pilot geriatric cohort, higher miR-132, miR-545, and miR-34a were associated with lower MMSE scores after adjustment. The effect sizes were small and remained uncertain in bootstrap analyses. These findings are exploratory and hypothesis-generating, require replication in larger, better-powered consecutive cohorts, and do not establish diagnostic, prognostic, or standalone clinical biomarker utility.

### Declarations

AI-assisted editing disclosure: OpenAI ChatGPT (GPT-5.5 Thinking; https://chatgpt.com/) was used to support technical editing, grammar and typo correction, clarity improvements, formatting checks, and preparation of administrative submission wording. It was not used to generate or analyze data, conduct statistical analyses autonomously, determine eligibility, interpret laboratory results, or draw scientific conclusions. All AI-assisted text was reviewed, edited, and verified by the authors, who take full responsibility for the final manuscript.

## Supporting information

S1 Data De-identified participant-level minimal dataset underlying the reported analyses. (XLSX)

S2 Data Dictionary. Variable names, definitions, units, and coding for S1 Data.
(XLSX)

S1 Table Non-detect handling and winsorization summary by miRNA. (DOCX)

S2 Table Secondary threshold-defined impairment models for individual miRNAs. (DOCX)
