# Supplementary material for: Individual circulating microRNAs and continuous cognitive performance in a mixed inpatient-outpatient geriatric cohort: A cross-sectional pilot study
Source: PLoS One. 2026 Sep 11;21(9):e0357295. doi: 10.1371/journal.pone.0357295 (PMC13567804; doi:10.1371/journal.pone.0357295)
Supplement: Supplementary file 1 [file pone.0357295.s003.docx]

S3 Table. Non-detect handling and winsorization summary by miRNA

Reactions with Cp values >=39 or no amplification were classified as non-detects. Counts for non-detect handling are final analytic participant duplicate-reaction counts from the 63-person analytic cohort, with 126 duplicate reactions per miRNA (63 participants x 2 duplicate reactions), excluding blank/control wells. Participant-level expression values were calculated from available valid target/cel-miR-39-normalized duplicate ratios; non-detect reactions were excluded rather than substituted.

| miRNA | Model-available n | Final analytic participant duplicate reactions with no amplification or Cp >=39 classified as non-detect, n/126 | Winsorized low, n | Winsorized high, n |
| --- | --- | --- | --- | --- |
| miR-146a | 61 | 5 | 0 | 3 |
| miR-181a | 60 | 5 | 0 | 2 |
| miR-34a | 61 | 39 | 0 | 2 |
| miR-545 | 55 | 39 | 0 | 1 |
| miR-125b | 61 | 8 | 0 | 2 |
| miR-532 | 61 | 38 | 0 | 1 |
| miR-132 | 61 | 44 | 0 | 1 |
| miR-181c | 62 | 48 | 0 | 1 |

Note: If one duplicate amplified and passed quality control, the participant-level value could still be available for modelling from the valid duplicate ratio. Participant-level miRNA values remained missing only when no valid normalized duplicate ratio was available after quality control. Winsorization was applied using the pre-winsorization mean +/-3 sample SD for each miRNA. No values were clipped at the lower bound; high-end values were clipped as shown. Final z-standardized values may exceed +/-3 because z-standardization was performed after winsorization. The public S1 expression variables represent the final post-winsorization participant-level cel-miR-39-normalized expression values; the corresponding z-standardized variables were calculated from these post-winsorization values.
