# Supplementary material for: Individual circulating microRNAs and continuous cognitive performance in a mixed inpatient-outpatient geriatric cohort: A cross-sectional pilot study
Source: PLoS One. 2026 Sep 11;21(9):e0357295. doi: 10.1371/journal.pone.0357295 (PMC13567804; doi:10.1371/journal.pone.0357295)
Supplement: Supplementary file 2 [file pone.0357295.s004.docx]

**S4 Table. Secondary threshold-defined impairment models for individual miRNAs**

Adjusted logistic-regression estimates are reported for each individual cel-miR-39-normalized miRNA per 1-standard-deviation higher expression level. Models were adjusted for age, sex, years of education, and recruitment setting. The sample size and event count correspond to the complete-case dataset available for each miRNA-outcome-covariate model. FDR q values were calculated using the Benjamini-Hochberg procedure within each eight-test outcome family. Threshold-defined outcomes were based on screening-test cut-offs and should not be interpreted as adjudicated clinical diagnoses. This table is limited to individual-marker logistic-regression estimates; it does not evaluate composite panels, AUC/ROC discrimination, calibration, or decision-curve performance.

| **Outcome** | **miRNA** | **n** | **Events** | **OR per 1 SD (95% CI)** | **p** | **FDR q** |
| --- | --- | --- | --- | --- | --- | --- |
| CI-MMSE | miR-132 | 61 | 14 | 1.85 (0.89 to 3.84) | 0.097 | 0.776 |
| CI-MMSE | miR-545 | 55 | 12 | 1.75 (0.74 to 4.16) | 0.202 | 0.807 |
| CI-MMSE | miR-34a | 61 | 15 | 1.28 (0.65 to 2.54) | 0.474 | 0.907 |
| CI-MMSE | miR-532 | 61 | 15 | 1.27 (0.64 to 2.53) | 0.498 | 0.907 |
| CI-MMSE | miR-125b | 61 | 14 | 1.03 (0.54 to 1.98) | 0.931 | 0.931 |
| CI-MMSE | miR-146a | 61 | 14 | 1.18 (0.62 to 2.27) | 0.614 | 0.907 |
| CI-MMSE | miR-181a | 60 | 13 | 1.15 (0.59 to 2.26) | 0.680 | 0.907 |
| CI-MMSE | miR-181c | 62 | 15 | 0.92 (0.50 to 1.71) | 0.795 | 0.909 |
| Dementia-MMSE | miR-132 | 61 | 6 | 2.15 (0.94 to 4.87) | 0.068 | 0.101 |
| Dementia-MMSE | miR-545 | 55 | 7 | 5.02 (0.98 to 25.87) | 0.054 | 0.101 |
| Dementia-MMSE | miR-34a | 61 | 7 | 3.78 (1.03 to 13.93) | 0.046 | 0.101 |
| Dementia-MMSE | miR-532 | 61 | 7 | 2.43 (0.83 to 7.06) | 0.104 | 0.104 |
| Dementia-MMSE | miR-125b | 61 | 7 | 2.21 (0.87 to 5.60) | 0.095 | 0.104 |
| Dementia-MMSE | miR-146a | 61 | 7 | 2.02 (0.93 to 4.37) | 0.074 | 0.101 |
| Dementia-MMSE | miR-181a | 60 | 7 | 2.68 (0.93 to 7.68) | 0.067 | 0.101 |
| Dementia-MMSE | miR-181c | 62 | 7 | 2.88 (0.89 to 9.28) | 0.076 | 0.101 |
| CI-ACE | miR-132 | 61 | 22 | 1.52 (0.78 to 2.99) | 0.220 | 0.540 |
| CI-ACE | miR-545 | 55 | 17 | 2.61 (0.86 to 7.95) | 0.091 | 0.540 |
| CI-ACE | miR-34a | 61 | 23 | 1.39 (0.68 to 2.85) | 0.364 | 0.583 |
| CI-ACE | miR-532 | 61 | 23 | 1.29 (0.64 to 2.60) | 0.481 | 0.641 |
| CI-ACE | miR-125b | 61 | 22 | 1.03 (0.54 to 1.97) | 0.918 | 0.918 |
| CI-ACE | miR-146a | 61 | 22 | 1.08 (0.57 to 2.04) | 0.815 | 0.918 |
| CI-ACE | miR-181a | 60 | 21 | 1.53 (0.72 to 3.23) | 0.270 | 0.540 |
| CI-ACE | miR-181c | 62 | 23 | 1.51 (0.77 to 2.97) | 0.232 | 0.540 |
| Dementia-ACE | miR-132 | 61 | 13 | 1.72 (0.85 to 3.50) | 0.131 | 0.462 |
| Dementia-ACE | miR-545 | 55 | 13 | 2.16 (0.82 to 5.67) | 0.117 | 0.462 |
| Dementia-ACE | miR-34a | 61 | 14 | 1.38 (0.70 to 2.72) | 0.357 | 0.477 |
| Dementia-ACE | miR-532 | 61 | 14 | 1.32 (0.68 to 2.58) | 0.418 | 0.477 |
| Dementia-ACE | miR-125b | 61 | 14 | 1.36 (0.65 to 2.87) | 0.417 | 0.477 |
| Dementia-ACE | miR-146a | 61 | 14 | 1.50 (0.74 to 3.07) | 0.261 | 0.477 |
| Dementia-ACE | miR-181a | 60 | 13 | 1.73 (0.79 to 3.81) | 0.173 | 0.462 |
| Dementia-ACE | miR-181c | 62 | 14 | 1.13 (0.58 to 2.16) | 0.724 | 0.724 |
